# Microbial symbiosis and coevolution of an entire clade of ancient vertebrates: the gut microbiota of sea turtles and its relationship to their phylogenetic history

**DOI:** 10.1186/s42523-020-00034-8

**Published:** 2020-05-07

**Authors:** Titus Franciscus Scheelings, Robert J. Moore, Thi Thu Hao Van, Marcel Klaassen, Richard D. Reina

**Affiliations:** 1 School of Biological Sciences, Monash University, Wellington Rd, Clayton, Victoria 3800 Australia; 2 RMIT University School of Science, Bundoora West Campus, Plenty Rd, Bundoora, Victoria 3083 Australia; 3 Centre for Integrative Ecology, Deakin University, Waurn Ponds, Victoria 3216 Australia

**Keywords:** Evolution, Microbiota, Phylogeny, Sea turtle

## Abstract

**Background:**

The microbiota plays a critical role in host homeostasis and has been shown to be a major driving force in host evolution. However, our understanding of these important relationships is hampered by a lack of data for many species, and by significant gaps in sampling of the evolutionary tree. In this investigation we improve our understanding of the host-microbiome relationship by obtaining samples from all seven extant species of sea turtle, and correlate microbial compositions with host evolutionary history.

**Results:**

Our analysis shows that the predominate phyla in the microbiota of nesting sea turtles was Proteobacteria. We also demonstrate a strong relationship between the bacterial phyla SR1 and sea turtle phylogeny, and that sea turtle microbiotas have changed very slowly over time in accordance with their similarly slow phenotypic changes.

**Conclusions:**

This is one of the most comprehensive microbiota studies to have been performed in a single clade of animals and further improves our knowledge of how microbial populations have influenced vertebrate evolution.

## Background

Residing on and within most metazoan species is a diverse and complex metropolis of microorganisms (viruses, bacteria, fungi and protozoans) known collectively as the microbiota and the occupation of higher organisms by prokaryotic and eukaryotic colonists has been a key factor in driving evolution and radiation of life on Earth [1, 2]. Hosts and their microbiotas form a mutualistic holobiont so intertwined that the amalgamation of these distinct organisms is often referred to as a holobiome, and their combined genetic material collectively defined as the hologenome [3, 4]. This multigenomic microcosm has become so integral to animal homeostasis that it can no longer be considered separate from the individual. Thus, it has been proposed that animals represent a vastly intricate biological ‘super organism’ in which a proportion of the physiological function is derived from microbial activity [5]. The microbiota has been shown to be a heritable trait in a range of vertebrates [6–11], and there is evidence to suggest that its composition and function is probably influenced by host phylogeny [12, 13], with co-evolution of the host and microbiota a critical process in shaping metazoan life [3, 4]. Ultimately, it appears that a bidirectional interaction of host physiology and gut microbiota over evolutionary time is responsible for determining host dietary niche and adaptation [12]. Understanding these relationships using a multi-disciplinary approach by combining microbiological techniques with phylogenetic analyses, is fundamental to exploring the origins of complex, multicellular organisms. Investigating closely-related species with clearly defined phylogenies is extremely informative, because analysis is not confounded by vast expanses of evolutionary periods with multiple missing links. Thus, evolutionarily old taxa with relatively simple phylogenetic histories are highly-desirable candidates for such an investigation. Sea turtles represent an excellent group of species to study in this context, because all extant species, including their phylogeny, are well described [14], and they originate from an approximately 200 million year old lineage that has gone through intermittent periods of slow and intermediate evolution and diversification [15], meaning that any described relationships are robust and profound. Exploring the bidirectional interplay of evolutionary forces acting on the host-microbiota relationship is an important step in comprehending the origins of metazoan physiology.

The major site for microbial inhabitation in animals is the gastrointestinal tract, where immense numbers of microorganisms confer myriad beneficial properties to their host. These complex interactions are an exciting and emerging area of evolutionary biology. Historically, the importance of microflora to non-human species has predominately focused on their role during digestion, particularly of complex carbohydrates in herbivorous animals. However, these investigations have begun to broaden with the realisation of the greater role that they play in the health and ecology of all species [2].

Investigations into the microbiota of reptiles, including sea turtles, are limited to studies describing microbial communities [16–29], factors that influence their composition [30–43], and how they affect host physiology [8, 9, 44–46], but investigations into the influence of phylogenetic factors affecting microbiota composition in this taxon are rare. Irrespective of the potential host-phylogenetic signal in microbiota composition at a broad taxonomic level, it has been shown that at a more individual level, diet, captivity, geography, and feeding regime all influence the microbiota [9, 17, 32], and that fermenting bacteria are important for digestion in herbivorous species [47].

Sea turtles are among the most imperilled species on Earth and therefore a deeper understanding of their physiology is important to their conservation. The role that microbiota plays in the health, behaviour and physiology of humans and animals is undeniable, and its categorisation in marine turtles is the next important step in understanding how these species adapt to a changing environment. There are seven extant species of sea turtle, the leatherback turtle (*Dermochelys coriacea*), green turtle (*Chelonia mydas*), flatback turtle (*Natator depressus*), hawksbill turtle (*Eretmochelys imbricata*), loggerhead turtle (*Caretta caretta*), olive ridley turtle (*Lepidochelys olivacea*), and the Kemp’s ridley turtle (*Lepidochelys kempii*). With the exception of the flatback turtle, all are listed as threatened species by the IUCN with various levels of risk, while the flatback turtle is listed as ‘Data Deficient’, but is also likely to be threatened [48]. Marine turtles are highly evolved for a completely aquatic life, but, like almost all reptiles they are still tied to the terrestrial environment for egg-laying [14]. The majority of sea turtle species are scattered unevenly throughout all three tropical oceans, with the exception of the flatback and Kemp’s ridley turtles, which have relatively restricted distributions [14]. Additionally, the leatherback turtle is a more cold adapted species with a more cosmopolitan distribution, and may be found occupying waters at higher latitudes than the other species [14]. Sea turtle diets vary remarkably over life stage, and among species [49]. For example, the leatherback feeds primarily on gelatinous zooplankton for its entire existence, while the green turtle is predominately omnivorous during the oceanic phase of its life, but then undergoes an ontogenic dietary shift to herbivory as it transitions to neritic (nearshore) habitats later in life [49], resulting in a discernible shift in the bacterial communities during this phase of their life [33]. Sea turtles are unique among the Reptilia, in that they are the only members that undergo long-distance migrations, rivalling those of other vertebrate species [50], and during this time they typically do not forage [51], which may have an effect on their microbiota [17, 52–56].

How the microbiota has contributed to the evolutionary history of sea turtles, and the extraordinary physiological adaptations of these species has been little studied, and as a result, we attempted to address this deficit by examining the microbial populations of the world’s sea turtle species. In this investigation we present the most comprehensive data on the microbiota of sea turtles that has been compiled to date. Furthermore, this is one of the most complete microbiota studies to have been conducted in any taxa, because we obtained samples from an entire clade of the evolutionary tree and we were able to explore some of the phylogenetic relationships that exist between sea turtles and their microbiotas. The aims of this investigation were to categorise the microbiota composition of the world’s sea turtle species, to explore any relationships between sea turtle phylogeny and microbiota composition, and to examine how sea turtle microbiotas have changed over evolutionary time.

## Results

Samples were collected from 120 turtles, with sequencing results obtained in all but three, in which failure of DNA extraction meant that results were not obtained for these individuals (Table 1 and Additional file 1).

**Table 1:** Summary of sequencing results

Species	Number Sampled	Number successfully sequenced	Total Sequences	Average sequences per sample
**Leatherback**	18	17	693,932	40,819
**Green**	18	17	612,452	36,026
**Flatback**	17	17	147,317	8665
**Loggerhead**	20	20	555,204	32,659
**Hawksbill**	20	20	27,986	1646
**Olive ridley**	10	9	152,007	16,889
**Kemp’s ridley**	20	20	212,631	12,507

**Fig. 1:**
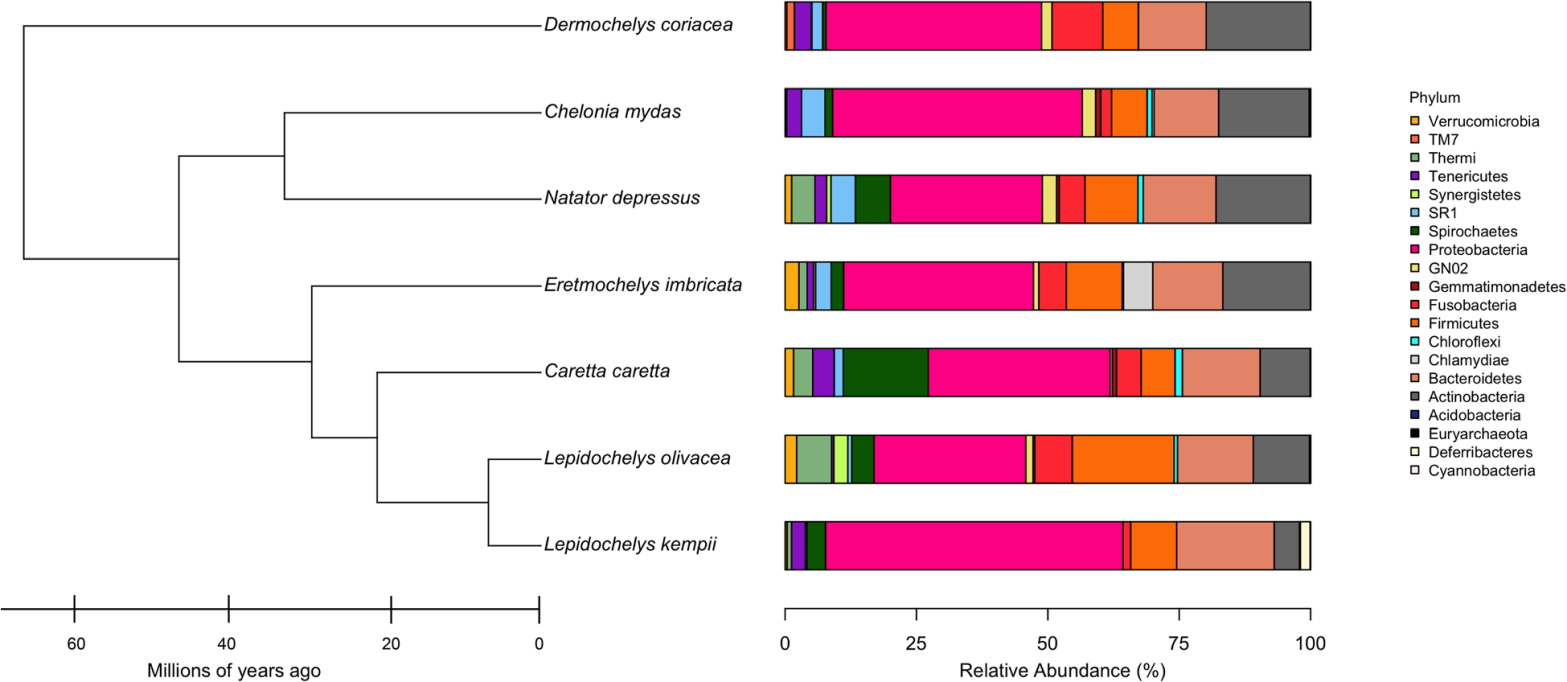
Relative abundance of bacterial phyla in each sea turtle species together with their phylogenetic tree

**Fig. 2:**
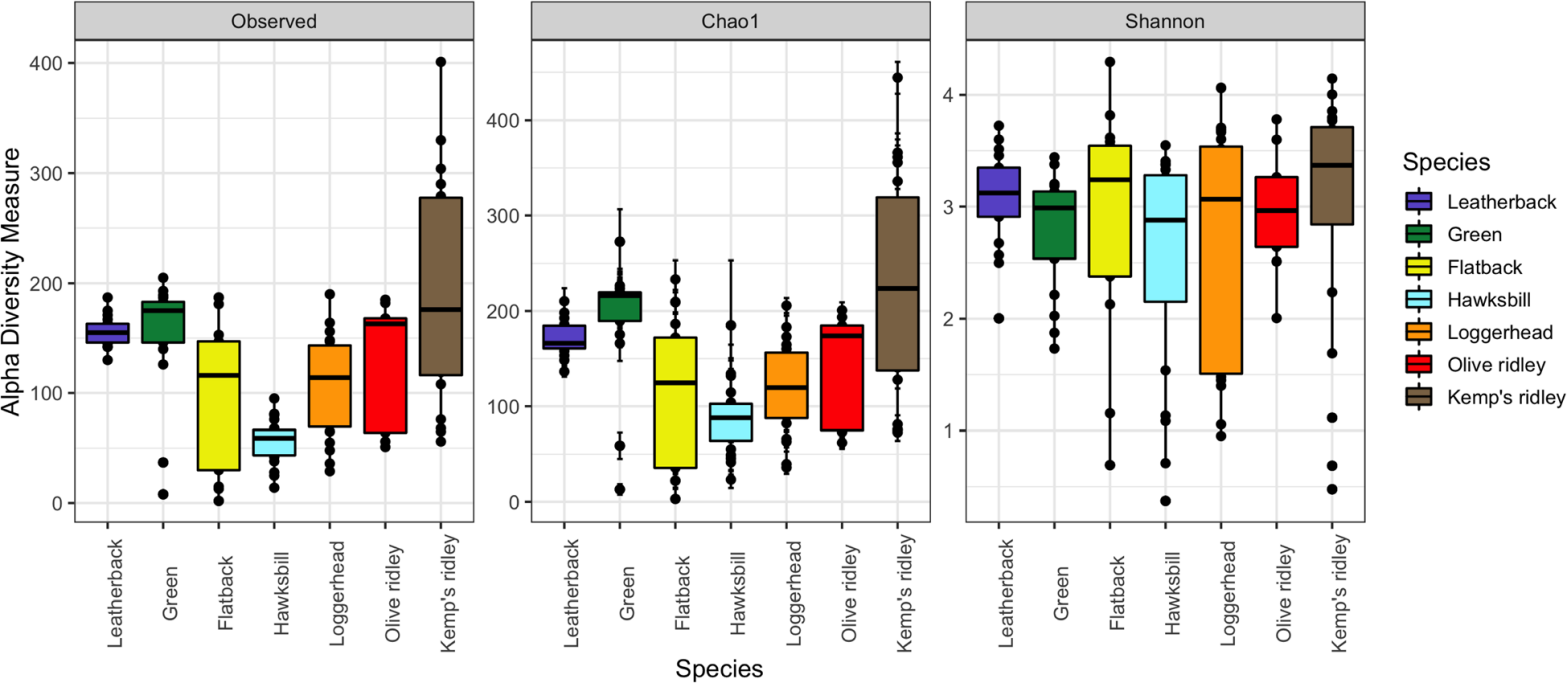
Alpha diversity measures across all species. Individual points and brackets represent the richness estimate and the theoretical standard error range associated with that estimate, respectively. Within each panel, the samples are organized into species, and a boxplot is overlaid on top of this. The mean microbial diversity estimate using Shannon’s diversity index did not differ significantly among all samples (*p* = 0.28). However, there were significant differences between samples as measured by Observed (*p* < 0.001) and Chao1 (p < 0.001)

**Fig. 3:**
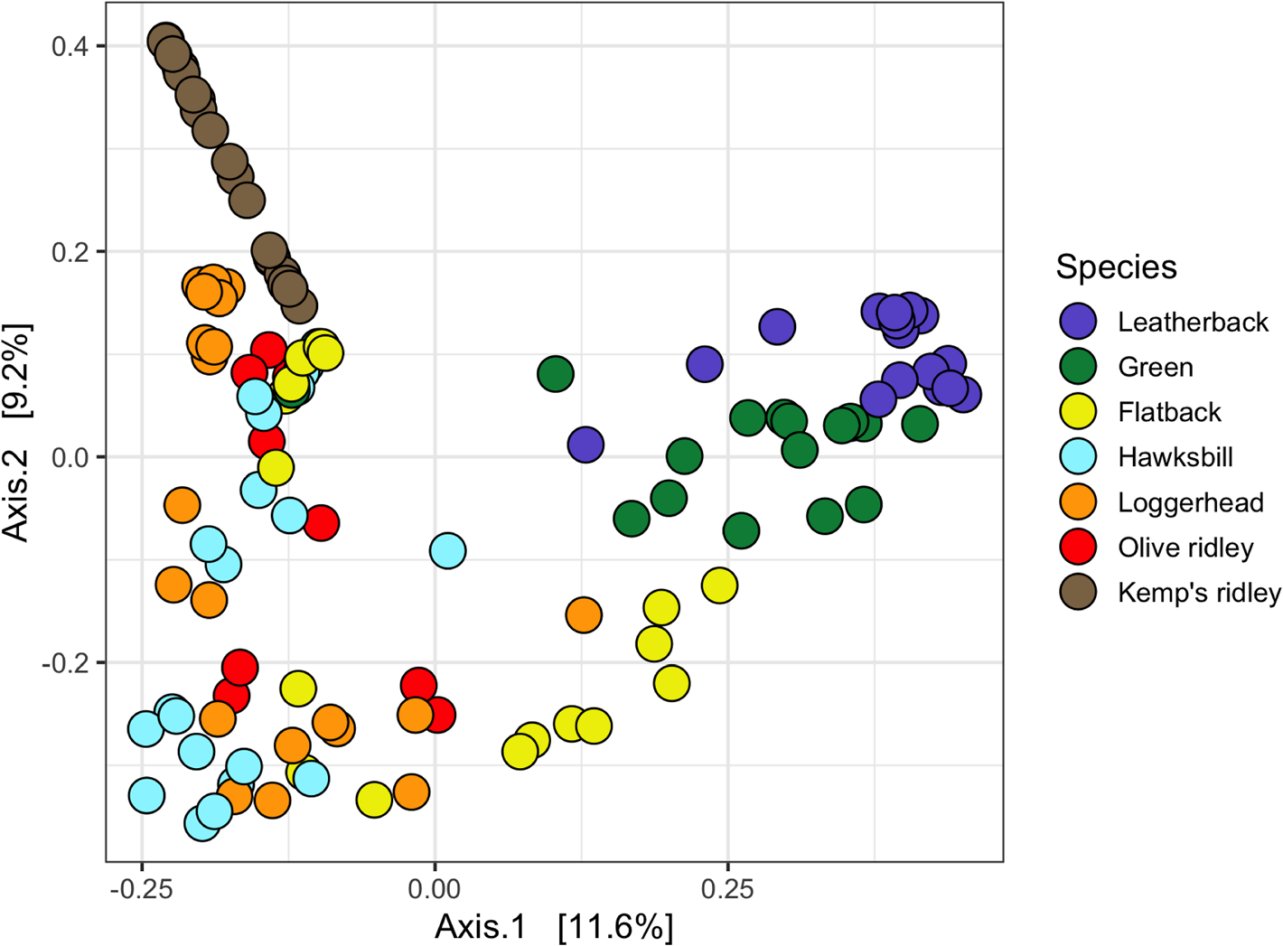
Principle co-ordinate analysis (PCoA) of Bray-Curtis distance showing functional microbial diversity across all species. Significant differences for microbiota composition existed between all species (R^2^ = 0.29, *p* = 0.001). For pairwise comparisons between species see Table S4

The taxonomic summary of microbial components from all samples yielded a total of 20 bacterial phyla, 36 classes, 63 orders, 122 families, 202 genera, and 362 OTUs (Fig. 1 and Additional file 1). The predominant bacterial phyla were Proteobacteria, Bacteroidetes, Actinobacteria, and Firmicutes (Fig. 1 and Additional file 1), while the phyla Euryarchaeota, Deferribacteres, and Cyanobacteria were only seen in Kemp’s ridley turtles. The prevalence of the phylum SR1 (Asoconditabacteria) was greatest in flatback and green turtles, intermediate in hawksbill and leatherbacks, and lowest in loggerhead and ridley turtles (Fig. 1 and Additional file 1). Analysis of alpha diversity revealed that there were significant differences between Observed OTUs (χ^2^ = 45.83, df = 6, *p* < 0.001) (Fig. 2 and Additional file 2), and in species richness between samples as measured by Chao1 (χ^2^ = 43.28, df = 6, p < 0.001) (Fig. 2 Additional file 2), but not in Shannon diversity (χ^2^ = 7.48, df = 6, *p* = 0.28) (Fig. 2 Additional file 2). These observations were consistent when all samples were analysed in entirety, and also for pairwise comparisons (Additional file 3). For beta diversity, we detected clustering patterns for the more ancient species (leatherback and green turtles), as well as the most modern species, Kemp’s ridley (Fig. 3). For the remaining species there was overlap of microbiota compositions (Fig. 3). However, Adonis analysis of the PCoA plot revealed that there was a significant difference between all species when analysed together (df = 6, SS_T_ = 15.42, MS = 2.57, f.model = 7.72, R^2^ = 0.29, *p* = 0.001), and similarly, when pairwise comparisons were made between all combinations of species, significant differences in microbiota compositions existed for all species combinations (Additional file 4).

**Fig. 4:**
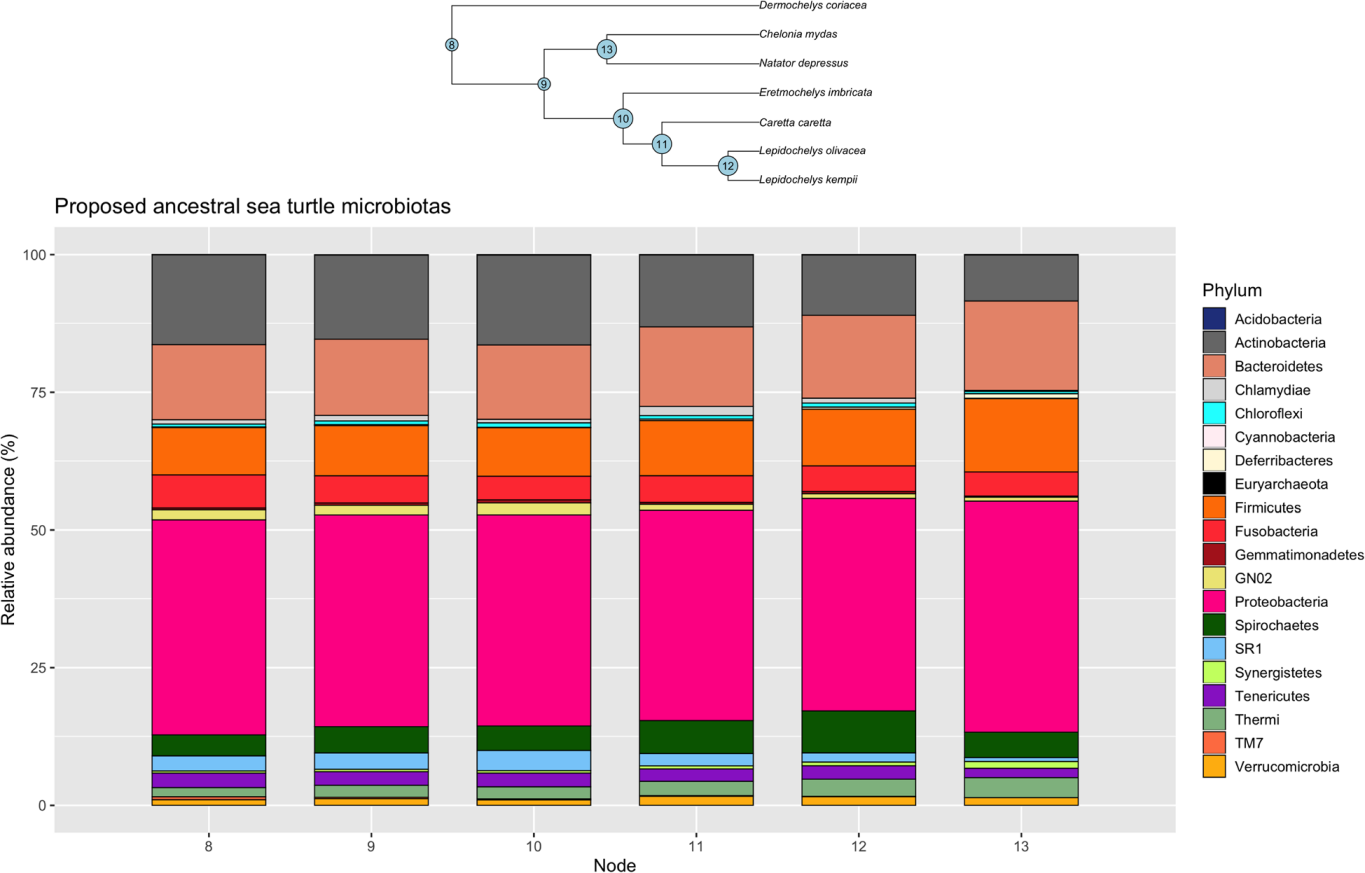
Proposed microbiotas for extinct sea turtle species with phylogenetic tree. Numbered nodes represent extinct ancestors on the sea turtle phylogenetic tree

We did not discover a phylogenetic signal for any alpha diversity measure of microbiota composition for any of the species in this investigation (Table S6 and Additional files 4, 5 and 6). When microbiota composition was analysed in concert with evolutionary history, we found that the bacterial phyla SR1, GN02 (Gracilibacteria) and Actinobacteria had a phylogenetic signal for both Moran’s *I* and Abouheif’s C_mean_ calculations (Table S7 and Additional files 7, 8 and 9). However, when these phyla were further examined using Pagels λ and Blomberg’s *K*-statistic, it was discovered that only SR1 (λ = 1.06, LogL = − 11.67, LogL0 = − 13.20, *p* = 0.08; *K* = 1.28, *p* = 0.02) was correlated to sea turtle phylogeny (GN02: λ = 0.62, LogL = − 9.38, LogL0 = − 9.67, *p* = 0.44; *K* = 0.69, *p* = 0.28; Actinobacteria: λ = 0.69, LogL = − 21.0, LogL0 = − 21.36, p = 0.4; *K* = 0.9, *p* = 0.14). The relative abundance of SR1 was greatest in green and flatback turtles, and lowest in olive ridley turtles (Fig. 1). Finally, we reconstructed extinct sea turtle microbiotas (numbered nodes (8–13) on the phylogenetic tree (Fig. 4)), to examine how these have changed over the course of sea turtle evolution. We found that most bacterial phyla have been relatively stable over time, with the exception of Actinobacteria, which decreased in relative abundance as sea turtles evolved, and Spirochaetes, which went through a period of increase between nodes 1–5, but then decreased from nodes 5–6. The phylum Thermi showed a steady increase across nodes as time progressed (Fig. 4).

## Discussion

In this investigation we present the most comprehensive data on the microbiota composition of sea turtles to date, and demonstrate a clear link between microbiota composition and sea turtle phylogeny. Furthermore, this is one of the only studies in wild animals to have obtained samples from all extant species of an entire clade of the evolutionary tree. This enabled us to perform a detailed evaluation of the phylogenetic signal that exists between host and microbiota. We showed that microbiota composition differs among all sea turtle species, but in all species, the predominant bacterial phylum in nesting turtles was Proteobacteria. In an investigation into the microbiota of juvenile green turtles from Florida, Proteobacteria was also the major phylum dominating samples [33]. In contrast, the microbiota of juvenile green turtles from coastal areas of Brazil are co-dominated by the phyla Bacteroidetes and Firmicutes, with the investigators speculating that Proteobacteria increased in abundance in three individual turtles in this investigation in response to anthropogenic factors [38]. Similarly, in a study conducted on green turtles from the Great Barrier Reef, Firmicutes was the most common phylum isolated from healthy individuals, but in sick turtles Proteobacteria was the dominate phylum [57]. There are also reports of Firmicutes dominating the microbiota of stranded loggerhead turtles [18, 37], but these investigations are confounded because samples were collected from sick individuals, and in many cases there were delays between when the turtle was rescued and when samples were collected. Given that both captivity [58–62] and health [63] have both been shown to affect the microbiota of individuals, these results should be interpreted with caution as they are unlikely to represent the normal gut microbiota. In our investigation, all turtles sampled were wild and apparently healthy, and some had been sourced from extremely remote locations with no human habitation (e.g. Rosemary Island and Tiwi Islands), so we think that the likelihood of anthropogenic or other factors influencing our results is low. Furthermore, given that Proteobacteria was overwhelmingly the predominate phylum in all sea turtle species, and the strong phylogenetic relationship between microbiota and sea turtle species, we think that in nesting animals (i.e. animals with prolonged periods of inappetence), any microbiota in which Proteobacteria is not the most abundant phylum represents an atypical gut flora.

In comparison to other taxa, there are few studies on the microbiota of wild reptiles, and the relative abundance of specific bacterial phyla in herpetofauna appears to vary greatly. For example, Firmicutes was the dominant phylum identified in anoles (*Anolis* sp.) [64], Galápagos tortoises (*Chelonoidis nigra*) [23], gopher tortoises (*Gopherus polyphemus*) [19], green iguanas (*Iguana iguana*) [65], Galápagos land iguanas (*Conolophus subcristatus*) [23], marine iguanas (*Amblyrhynchus cristatus*) [23], and the montane iguana species *Liolaemus parvus*, *Liolaemus ruibali*, and *Phymaturus williamsi* [9]. Such findings have led some researchers to believe that reptile microbiotas resemble that of mammals [21, 32], but in a wide-ranging investigation into squamate microbiotas, in which individuals representing 22 squamate families were sampled, Proteobacteria was the predominate phylum, and supports a hypothesis that the reptile gut microbiota is similar to that of birds [66]. However, in the single reptile study conducted in the species most closely related to birds, the archosaurian American alligator (*Alligator mississippiensis*), gut microbiota was overwhelmingly dominated by the phylum Fusobacteria [17]. Furthermore, the results of our investigation are similar to those seen in fish, in which the predominate phyla are Proteobacteria, Bacteroidetes, Actinobacteria, and Fusobacteria [67]. These discrepancies highlight the difficulties associated with making any assumptions on microbial assembly between taxa based solely on compositional data without incorporating any phylogenetic techniques in the analysis.

Unlike their microbiotas, the natural histories of sea turtles differ widely among the seven extant species. Leatherbacks are largely oceanic-pelagic throughout their life-history, and have the most specialised diet, feeding almost entirely on an array of dense gelatinous zooplankton [49]. Their large size means that they are more cold-adapted than other species, allowing them to traverse through the boreal waters that act as a barrier to other warm-water adapted turtles [68]. Green turtles have an oceanic-neritic developmental pattern [69], are found in tropical and temperate waters worldwide [68], and consume a variety of seagrass, marine algae, and invertebrates [49, 70]. Like green turtles, loggerheads also have an oceanic-neritic developmental pattern and prefer temperate to tropical waters [69]. However, unlike green turtles, they are largely carnivorous, feeding on a wide array of prey items including Hydrozoa, Bivalvia, Cephalopodia, Porifera, Scyphoza, Bryozoa, Gastropoda, Polychaeta, Maxillopoda, Malacostraca, Insecta, Holothuroidea, Echinoidea, Anthozoa, Actinopterygii, and occasional plant material [49]. Evidence suggests that hawksbills also have an oceanic-neritic developmental pattern [69], with a preference for tropical waters [69]. Although primarily carnivorous, the composition of prey items varies among populations of hawksbills, with some having a preference for sponges, while others feed predominately on corals [49]. The flatback turtle has a completely neritic life history [69], residing within the tropical waters of the Australian continental shelf [71]. Detailed investigations into their diet are lacking, but it is presumed that they are carnivorous [49]. The olive ridley turtle is predominately oceanic for the duration of its life [69], and is found in both temperate and tropical waters, but most feeding probably occurs in warm water and they are likely omnivorous [49, 68]. Finally, Kemp’s ridleys have an oceanic-neritic developmental pattern [69], have a preference for tropical waters, and as adults are primarily carnivorous ingesting a range of molluscs, fish, jellyfish and gastropods [49]. Given the vast array of niche occupancy and dietary preferences of sea turtles, some with significant overlap between species, it is likely that the similarities seen in relative abundance of the major bacterial phyla are driven by their shared evolutionary history rather than dietary and ecological factors. The similarities seen between gut microbiotas in nesting female sea turtles is remarkable, since there is nearly 100 million years of evolution separating the most ancient species, the leatherback, and the most modern species, the Kemp’s ridley [15]. Such conservation of community similarity may be an indication that specific combinations of bacterial phyla are fundamental to sea turtle normal function, but this remains to be demonstrated in any taxa.

Based on the results of our study, it appears that gut microbial composition and sea turtle phylogeny are intrinsically linked, and it is likely that a process of co-evolution exists between host and microbial community composition. Microbes have been identified as a key driver of vertebrate evolution [2], and more recent research has focused on the role that phylogeny may play in convergence of microbiotas in some species [72]. Investigations in primates suggest that evolutionary trends in host physiology are more important than dietary niche in determining gastrointestinal microbiota [12], which is supported by this research. For example, previous investigations into sea turtle microbiotas have speculated that the high proportion of Firmicutes found in some green turtle samples may be due to this bacterial phylum’s ability to break down plant-derived polysaccharides [57] and thus facilitate digestion of a cellulose-rich food such as the seagrass species that form the primary diet item of green turtles. However, we found that green turtles, along with loggerheads, had the lowest relative abundance of Firmicutes of all the sea turtle species, suggesting that this phylum may not be as important for cellulose digestion in herbivorous reptiles as previously reported. However, it should be remembered that since the animals sampled in our investigation were not feeding, it is possible that there may have been a shift away from phyla important for digestive function and thus they were underrepresented relative to what would be expected in feeding animals. Nevertheless, there are many examples in vertebrates, both terrestrial and aquatic, and from disparate branches of the evolutionary tree, where Firmicutes are lower in abundance than other phyla in herbivorous species [73–81]. Therefore, in the absence of specific functional testing, caution should be applied when making assumptions on functionality of the microbiota within a species, especially where phylogeny has not been considered as a component of the analysis. The results of this analysis show that extant sea turtle microbiotas have changed very little over the course of nearly 70 million years of evolution, despite the phenotypic changes that have occurred in their hosts, and this may be an indication that certain combinations of microbes are fundamental to specific aspects of all sea turtle physiology, regardless of differences in natural history between species.

We showed that the bacterial phylum SR1 was strongly linked to sea turtle phylogeny. The candidate phylum SR1 (Absconditabacteria), includes ubiquitous organisms found in marine and terrestrial high-temperature environments, fresh-water lakes, subsurface aquifers, and animals [82, 83]. There are no cultured representatives of SR1, with all current knowledge on their presence and diversity obtained from genomic sequencing [84, 85]. They have a predilection for sulphur-rich and oxygen-limited environments, suggesting a potential microaerophilic, sulphur-based metabolism, and in general, environmental and animal-derived SR1 species appear to cluster differently [83, 86]. SR1 is routinely found in a range of vertebrates, but is most commonly associated with H_2_S-related malodour and periodontal disease in humans [87, 88]. How SR1 is involved in sea turtle gastrointestinal function remains unknown at this stage. The role of the microbiota in shaping vertebrate phylogeny should be a focus for future investigations, and an effort must be made to sample as diverse an array of species as possible, spanning multiple clades of the evolutionary tree, so that these relationships can be further explored.

Using results obtained from extant turtles, we reconstructed possible microbiotas of extinct sea turtle species. This analysis showed that the composition of sea turtle microbiotas has not changed greatly over time, with only relatively minor fluctuations in the relative abundance of specific phyla responsible for observed differences. In contrast, the human microbiota has diverged rapidly from our closest relatives [89], and among populations of humans, continues to rapidly evolve [90]. Sea turtles are among the most ancient species on Earth, with the leatherback existing in its current form for nearly 70 million years [15], while modern humans first appeared around 350,000 to 260,000 years ago [91]. These seemingly marked differences in rate of evolution of microbiotas, may be reflective of broad differences in rates of evolution between taxa, and warrants further investigation.

A feature of our study was that we were restricted to sampling nesting females. This was because living males are difficult to obtain, and this can only be done in-water. Although this means that our analysis and interpretation is made on a particular subset of animals of the same sex, life stage and reproductive state for each species, it removes potentially confounding variation in the composition of microbiota associated with these traits and thus we think it strengthens our phylogenetic comparisons. It is likely that all of the females sampled in this investigation had not eaten for an extended period, as sea turtles may undergo long periods of fasting, particularly during breeding and migration [51, 92]. Periods of inappetence have been shown to affect the microbiotas of a range of vertebrates including humans [52], fish [53], bears [54], alligators [17], mice [55], penguins [56], and the Burmese python [32], however such changes are not consistent across all taxa, with fasting having negligible effects on the microbiota of geckoes [93]. How this may have influenced our results is unknown, but it may explain the differences in our results and those of other green turtles captured on the Great Barrier Reef [57], and future investigations should focus on obtaining samples from a range of age classes, sexes, and physiological states. Although we were not able to determine if fasting in nesting turtles affected microbial diversity, some authors propose that fasting samples represent the core microbial OTUs, with other OTUs fluctuating in number in response to post-prandial physiological changes [17, 32]. If, as hypothesised, diet has little effect on this core microbiota, then this strengthens the results of our phylogenetic analysis because our results have not been confounded by transient microbial species that might be associated with dietary variation.

## Conclusions

Our investigation represents the most comprehensive microbiota study to have been conducted in sea turtles to date, but most importantly, we were able to obtain samples from an entire clade of the evolutionary tree, which allowed us to perform a comprehensive phylogenetic analysis. While other investigations have sampled multiple species in the single study [22, 78, 81, 94, 95], the breadth of taxonomic coverage in these analyses was less comprehensive from a phylogenetic perspective. We were able to show that in nesting turtles, the microbiota is predominated by the phylum Proteobacteria and that the phylum SR1 is strongly linked to sea turtle phylogeny. Understanding the structure of microbial populations, and the complexities of the host-microbiome relationship, is the next critical step in managing threatened species populations. Furthermore, only by unravelling these mysteries can we truly understand the origins of life, and the forces that have shaped the diversity in form and function we see today.

## Methods

### Study populations

All samples were collected from adult female turtles as they nested during their respective breeding seasons. Flatback turtles (*n* = 17) were sampled from Port Hedland, Western Australia (20.3107° S, 118.5878° E) in November 2016. Green sea turtles (*n* = 18), were sampled from Heron Island, Queensland, Australia (23.4423° S, 151.9148° E) in January 2017. Loggerhead turtles (*n* = 20) were sampled from Mon Repos, Queensland, Australia (24.8059° S, 152.4416° E) in January 2017. Hawksbill turtles (n = 20) were sampled from Rosemary Island, Western Australia (20.2846° S, 116.3540° E) in October 2017. Olive ridley turtles (*n* = 10) were sampled from Tiwi Islands, Northern Territory, Australia (11.6969° S, 130.8779° E) in April 2018. Leatherback turtles (n = 18) were sample from Juno Beach, Florida, USA (26.5224° N, 80.315° E) in May 2018. Kemp’s ridley turtles (n = 20) were sampled from Playa Rancho Nuevo, Tamaulipas, Mexico (23.11° N, 97.46° W) in June 2019.

### Sample collection

For leatherback turtles we waited until the female was covering the nest and then we dug a channel in the sand behind the turtle so that a swab could be inserted into the cloaca while the animal was still in a ventral position. For all other species, we waited until they had finished laying and were returning to the ocean and the turtle was then flipped into a dorsal position for sample collection. In all turtles an equine uterine swab (Minitube, Smythesdale, Victoria, Australia) was inserted into the cloaca so that it entered the distal colon. We were confident of correct placement of the swab because they are 90 cm long and were inserted to a depth of at least 60 cm, which is much longer than the length of the cloaca based on the primary author’s experience with endoscopic examination of chelonian cloacas, [96]. These swabs were housed in a sterile sheath, the entire apparatus was inserted into the cloaca and the swab tip was extruded to collect the colonic sample when correct placement of the sheath had been achieved. The swab tip was then retracted back into the sheath prior to extraction to shield it from any environmental contamination. By using these swabs in this manner, we did not collect any negative control swabs as we were confident that there was no risk for contamination and samples represented true colonic samples. Turtles were then permitted to return to the ocean and then the tip of the swab was cut using a sterile wire cutter, placed into a sterile Eppendorf tube and sealed. Total sample collection time was approximately 10 min. The Eppendorf tube containing the swab was then immediately placed into a portable cool box filled with ice, and once back at the field station they were frozen at − 20 °C for approximately 3–4 days. Swabs were transported back to the laboratory using dry ice (− 78.5 °C), where they were stored at − 80 °C until extraction could take place approximately 1 week later.

### DNA extraction

DNA was extracted manually. In each Eppendorf tube, 500 μL of extraction buffer (20 mM EDTA, 0.1 M Tris, 1% CTAB, 56 mM NaCl, pH 8) was added so that swabs were completely covered. We then added 20 μL of Proteinase K (QIAGEN Proteinase K (10 ml(> 600 mAU/ml)) to each vial, along with 60 μL of 10% SDS. The mixture was then incubated at 55 °C overnight. The next day, 50 μL of 5 M NaCl and 500 μL of Phenol was added, and the tubes shaken until an emulsion was formed. They were then incubated at room temperature for 10 min, with intermittent mixing. The tubes were then centrifuged at 11,200 RCF for 10 min and the supernatant removed and added to a new tube containing 250 μL Phenol and 250 μL Chlorophorm:Isoamyl-Alcohol (24:1). The tubes were again centrifuged at 11,200 RCF for 10 min and the resultant supernatant added to a new tube containing 500 μL of Chlorophorm:Isoamyl-Alcohol. Once again, the tubes were centrifuged at 11,200 RCF for 10 min. The supernatant was then added to a new tube containing 3 M Sodium Acetate at a volume equal to 10% of the extraction solution. We then added 1 ml of ice-cold 99% ethanol to each test tube and then placed them into a freezer at − 20 °C for 1 h. The tubes were then centrifuged at 4 °C at 16,128 RCF for 10 min. The fluid in the test tube was then removed with a glass pipette and 1 ml of ice-cold 70% alcohol was added. The tubes were centrifuged a final time at 4 °C at 16,128 RCF for 5 min. After centrifugation the alcohol was removed and the lids left off the tubes to allow the DNA pellet to dry. Once dried, 25 μL of 1 x TE was added to each tube and the extracted DNA was stored at − 20 °C until amplicon sequencing could take place.

### 16S rRNA gene amplicon sequencing

The V3-V4 region of 16S rRNA genes were amplified with forward primer 5′ ACTCCTACGGGAGGCAGCAG 3′ and reverse primer 5′ GGACTACHVGGGTWTCTAAT 3′ using Q5 high fidelity polymerase (New England Biolabs). Sequencing was performed on an Illumina MiSeq system (2 × 300 bp) by the method of Fadrosh DW, Ma B, Gajer P, Sengamalay N, Ott S, Brotman RM and Ravel J [97].

### Data processing

Sequence data was analysed using QIIME version 1.9.1 [98] using default parameters and a Phred quality threshold of > 20. The UCLUST algorithm [99] was used to pick OTUs at 97% sequence identity and a Biome table was produced. Potentially chimeric sequences were identified using Pintail [100]. Blast was used to assign taxonomy against the Greengenes database [101] and QIIME version 1.9.1 defaults. Additional assignment of taxonomy was performed using a command line version of BLASTN [102] against the NCBI 16S Microbial database.

### Statistics and data analysis

Initial exploration of the Biome table data was performed using the online Calypso software [103]. Data was further analysed in R, utilising the package ‘phyloseq’ [104]. Alpha diversity was explored using Observed OTUs, Shannon index and Chao1 estimates. Alpha diversity was tested for normality using the Shapiro-Wilks test, with all metrics being non-normally distributed (Observed: W = 0.95, *p* < 0.001; Chao1: W = 0.96, *p* = 0.001; Shannon: W = 0.9, p < 0.001), and so comparisons between groups were first made using the Kruskal-Wallis test, and then paired comparisons between groups were made using the pairwise Wilcoxon rank sum test with (Holm) *p*-values that were adjusted for multiple comparisons. Beta diversity was investigated using principle co-ordinate analysis (PCoA) (Bray-Curtis) and Adonis tests to compare all species, and then pairwise comparisons were made between all combinations of species with Holm correction of p-values for multiple comparisons.

To test phylogenetic signal related to microbial composition in sea turtles, we first obtained genetic sequence data for the sea turtle species from NCBI (www.ncbi.nlm.nih.gov), and used these to construct a phylogenetic tree, with branch lengths, using the online database “Interactive Tree of Life” [105]. We then compiled the microbial data for all species into a Microsoft Excel spread sheet, and imported both the tree and microbiota data into R, and used the package ‘phytools’ to explore the effects of phylogeny on microbiota composition [106]. We used Moran’s *I*, and Abouheif’s C_mean_ to determine if specific bacterial phyla had a phylogenetic signal and whether or not there was a phylogenetic signal in alpha diversity. Where a significant result was found, this was further confirmed using Blomberg’s *K*-statistics, and Pagel’s λ for the individual bacterial phylum [107]. Finally, we reconstructed probable microbiota compositions of extinct sea turtle species at each of the nodes in our phylogenetic tree. For all statistical analyses significance was accepted if *p* > 0.05.

## Supplementary information

**Additional file 1: Table S1.** Sequencing results for all sea turtle species in this investigation

**Additional file 2: Table S2.** Pairwise Wilcoxon rank sum test comparisons between species for Observed OTUs. Numbers represent corrected (Holm) *p*-values for multiple comparisons. Significant values are indicated by bold text. **Table S3.** Pairwise Wilcoxon rank sum test comparisons between species for Chao1. Numbers represent corrected (Holm) p-values for multiple comparisons. Significant values are indicated by bold text. **Table S4.** Pairwise Wilcoxon rank sum test comparisons between species for Shannon index. Numbers represent corrected (Holm) *p*-values for multiple comparisons. No significance was observed between Shannon index for any inter-species comparisons

**Additional file 3: Table S5.** Pairwise Adonis comparisons of beta diversity for all combinations of microbiota composition in sea turtles. Both the p, and adjusted (Holm) p-values for multiple comparisons are reported. All pairwise comparisons were significantly different. For microbial composition of species and how they relate to other species refer to Fig. 3

**Additional file 4: Table S6**. Moran’s I and Abouheif’s C_mean_ calculations for alpha diversity and their correlation to sea turtle phylogeny. The observed value of I (Obs), is the expected value under the null hypothesis of no correlation. Positive values indicate that the data is spatially clustered in some way. Other values represented in this table include the standard-deviation of the observed I (Std.Obs), and the alternative hypothesis (alter) which has been set to “greater” meaning that the p-value is estimated as a number of random values equal to, or greater than the observed, + 1. No significance was detected for any diversity measure.

**Additional file 5: Fig. S1** Graphical representation of Moran’s *I* calculations for alpha diversity. Bars represent phylogenetic tree branches, the diamond represents the observed value, and the y-scale is frequency.

**Additional file 6: Fig. S2** Graphical representation of Abouheif’s C_mean_ calculations for alpha diversity. Bars represent phylogenetic tree branches, the diamond represents the observed value, and the y-scale is frequency.

**Additional file 7: Table S7.** Moran’s I and Abouheif’s C_mean_ calculations for bacterial phyla and their correlation to sea turtle phylogeny. Significant values are indicated by bold text.

**Additional file 8: Fig. S3** Graphical representation of Moran’s *I* calculations for microbial composition. Bars represent phylogenetic tree branches, the diamond represents the observed value, and the y-scale is frequency.

**Additional file 9: Fig. S4** Graphical representation of Abouheif’s C_mean_ calculations for microbial composition. Bars represent phylogenetic tree branches, the diamond represents the observed value, and the y-scale is frequency.

## Data Availability

All data not presented here or in the supplementary material have been submitted to The National Center for Biotechnology Information www.ncbi.nlm.nih.gov.

## Abbreviations

CTAB: Cetrimonium bromide
DNA: Deoxyribonucleic acid
EDTA: Ethylenediaminetetraacetic acid
GN02: Gracilibacteria
IUCN: International Union for Conservation of Nature
NCBI: National Centre for Biotechnology Information
OTU: Operational taxonomic unit
PCoA: Principal coordinate analysis
RCF: Relative centrifugal force
rRNA: Ribosomal ribonucleic acid
SDS: Sodium dodecyl sulfate
SR1: Asoconditabacteria
USA: United States of America
