# Supplementary material for: Microbial symbiosis and coevolution of an entire clade of ancient vertebrates: the gut microbiota of sea turtles and its relationship to their phylogenetic history
Source: Anim Microbiome. 2020 May 7;2:17. doi: 10.1186/s42523-020-00034-8 (PMC7807503; doi:10.1186/s42523-020-00034-8)
Supplement: Supplementary file 2 — Additional file 2: Table S2. Pairwise Wilcoxon rank sum test comparisons between species for Observed OTUs. Numbers represent corrected (Holm) p-values for multiple comparisons. Significant values are indicated by bold text. Table S3. Pairwise Wilcoxon rank sum test comparisons between species for Chao1. Numbers represent corrected (Holm) p-values for multiple comparisons. Significant values are indicated by bold text. Table S4. Pairwise Wilcoxon rank sum test comparisons between species for Shannon index. Numbers represent corrected (Holm) p-values for multiple comparisons. No significance was observed between Shannon index for any inter-species comparisons [file 42523_2020_34_MOESM2_ESM.docx]

**Additional File 2**

**Table S2.**

| Species | Flatback | Green | Hawksbill | Kemp's ridley | Leatherback | Loggerhead |
| --- | --- | --- | --- | --- | --- | --- |
| Green | 0.08 | - | - | - | - | - |
| Hawksbill | 0.82 | **<0.01** | - | - | - | - |
| Kemp's ridley | 0.08 | 1.00 | **<0.01** | - | - | - |
| Leatherback | 0.06 | 0.82 | **<0.01** | 1.00 | - | - |
| Loggerhead | 1.00 | **0.02** | **0.01** | 0.06 | **0.01** | - |
| Olive ridley | 1.00 | 1.00 | 0.08 | 0.96 | 1.00 | 1.00 |

**Table S3**

| Species | Flatback | Green | Hawksbill | Kemp's ridley | Leatherback | Loggerhead |
| --- | --- | --- | --- | --- | --- | --- |
| Green | **0.03** | - | - | - | - | - |
| Hawksbill | 1.00 | **<0.01** | - | - | - | - |
| Kemp's ridley | **0.03** | 1.00 | **<0.01** | - | - | - |
| Leatherback | 0.21 | **0.03** | **<0.01** | 0.52 | - | - |
| Loggerhead | 1.00 | **<0.01** | 0.29 | **0.02** | **<0.01** | - |
| Olive ridley | 1.00 | 0.21 | 0.31 | 0.23 | 1.00 | 1.00 |

**Table S4**

| Species | Flatback | Green | Hawksbill | Kemp's ridley | Leatherback | Loggerhead |
| --- | --- | --- | --- | --- | --- | --- |
| Green | 1.00 | - | - | - | - | - |
| Hawksbill | 1.00 | 1.00 | - | - | - | - |
| Kemp's ridley | 1.00 | 0.81 | 0.81 | - | - | - |
| Leatherback | 1.00 | 1.00 | 1.00 | 1.00 | - | - |
| Loggerhead | 1.00 | 1.00 | 1.00 | 1.00 | 1.00 | - |
| Olive ridley | 1.00 | 1.00 | 1.00 | 1.00 | 1.00 | 1.00 |
