# Supplementary material for: Microbial symbiosis and coevolution of an entire clade of ancient vertebrates: the gut microbiota of sea turtles and its relationship to their phylogenetic history
Source: Anim Microbiome. 2020 May 7;2:17. doi: 10.1186/s42523-020-00034-8 (PMC7807503; doi:10.1186/s42523-020-00034-8)
Supplement: Supplementary file 3 — Additional file 3: Table S5. Pairwise Adonis comparisons of beta diversity for all combinations of microbiota composition in sea turtles. Both the p, and adjusted (Holm) p-values for multiple comparisons are reported. All pairwise comparisons were significantly different. For microbial composition of species and how they relate to other species refer to Fig. 3 [file 42523_2020_34_MOESM3_ESM.docx]

**Additional File 3**

**Table S5**

| Pairs | df | SS_T_ | F.model | R^2^ | p | p.adjusted |
| --- | --- | --- | --- | --- | --- | --- |
| Leatherback vs Olive ridley | 1.00 | 2.59 | 10.36 | 0.30 | 0.001 | 0.021 |
| Leatherback vs Green | 1.00 | 2.13 | 8.41 | 0.21 | 0.001 | 0.021 |
| Leatherback vs Hawksbill | 1.00 | 3.89 | 13.37 | 0.28 | 0.001 | 0.021 |
| Leatherback vs Loggerhead | 1.00 | 3.80 | 13.21 | 0.27 | 0.001 | 0.021 |
| Leatherback vs Flatback | 1.00 | 2.91 | 10.29 | 0.24 | 0.001 | 0.021 |
| Leatherback vs Kemp's ridley | 1.00 | 4.25 | 14.62 | 0.29 | 0.001 | 0.021 |
| Olive ridley vs Green | 1.00 | 1.98 | 6.15 | 0.20 | 0.001 | 0.021 |
| Olive ridley vs Hawksbill | 1.00 | 1.56 | 4.31 | 0.14 | 0.001 | 0.021 |
| Olive ridley vs Loggerhead | 1.00 | 1.02 | 2.83 | 0.09 | 0.006 | 0.021 |
| Olive ridley vs Flatback | 1.00 | 1.39 | 3.85 | 0.14 | 0.001 | 0.021 |
| Olive ridley vs Kemp's ridley | 1.00 | 2.10 | 5.78 | 0.18 | 0.001 | 0.021 |
| Green vs Hawksbill | 1.00 | 2.92 | 8.59 | 0.20 | 0.001 | 0.021 |
| Green vs Loggerhead | 1.00 | 2.79 | 8.28 | 0.19 | 0.001 | 0.021 |
| Green vs Flatback | 1.00 | 1.69 | 5.02 | 0.14 | 0.001 | 0.021 |
| Green vs Kemp's ridley | 1.00 | 3.32 | 9.77 | 0.22 | 0.001 | 0.021 |
| Hawksbill vs Loggerhead | 1.00 | 1.89 | 5.20 | 0.12 | 0.001 | 0.021 |
| Hawksbill vs Flatback | 1.00 | 1.76 | 4.80 | 0.12 | 0.001 | 0.021 |
| Hawksbill vs Kemp's ridley | 1.00 | 3.02 | 8.22 | 0.18 | 0.001 | 0.021 |
| Loggerhead vs Flatback | 1.00 | 1.79 | 4.92 | 0.12 | 0.001 | 0.021 |
| Loggerhead vs Kemp's ridley | 1.00 | 3.07 | 8.40 | 0.18 | 0.001 | 0.021 |
| Flatback vs Kemp's ridley | 1.00 | 2.82 | 7.68 | 0.18 | 0.001 | 0.021 |
