# Supplementary material for: Microbial symbiosis and coevolution of an entire clade of ancient vertebrates: the gut microbiota of sea turtles and its relationship to their phylogenetic history
Source: Anim Microbiome. 2020 May 7;2:17. doi: 10.1186/s42523-020-00034-8 (PMC7807503; doi:10.1186/s42523-020-00034-8)
Supplement: Supplementary file 4 — Additional file 4: Table S6. Moran’s I and Abouheif’s Cmean calculations for alpha diversity and their correlation to sea turtle phylogeny. The observed value of I (Obs), is the expected value under the null hypothesis of no correlation. Positive values indicate that the data is spatially clustered in some way. Other values represented in this table include the standard-deviation of the observed I (Std.Obs), and the alternative hypothesis (alter) which has been set to “greater” meaning that the p-value is estimated as a number of random values equal to, or greater than the observed, + 1. No significance was detected for any diversity measure. [file 42523_2020_34_MOESM4_ESM.docx]

**Additional File 4**

**Table S6**

| Diversity Measure | Moran’s *I* | | | | Abouheif’s C_mean_ | | | |
| --- | --- | --- | --- | --- | --- | --- | --- | --- |
|  | Obs | Std.Obs | Alter | p | Obs | Std.Obs | Alter | p |
| Observed | -0.22 | -0.30 | greater | 0.56 | -0.22 | -0.25 | greater | 0.53 |
| Chao1 | -0.29 | -0.61 | greater | 0.68 | -0.29 | -0.57 | greater | 0.661 |
| Shannon | -0.14 | 0.12 | greater | 0.40 | -0.14 | 0.08 | greater | 0.42 |
