# Supplementary figures and images for: Microbial symbiosis and coevolution of an entire clade of ancient vertebrates: the gut microbiota of sea turtles and its relationship to their phylogenetic history
Source: Anim Microbiome. 2020 May 7;2:17. doi: 10.1186/s42523-020-00034-8 (PMC7807503; doi:10.1186/s42523-020-00034-8)

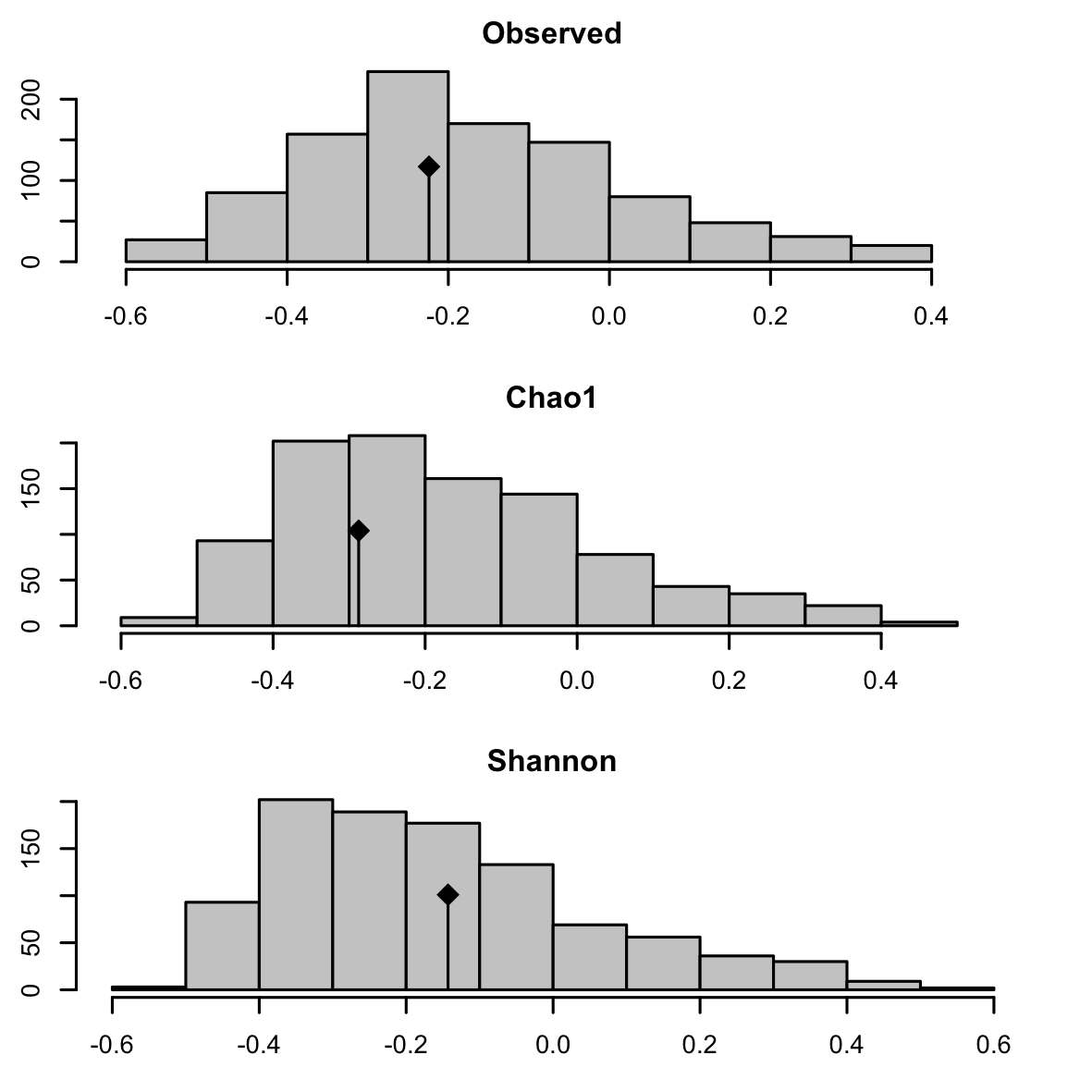

Supplement: Supplementary file 5 — Additional file 5: Fig. S1 Graphical representation of Moran’s I calculations for alpha diversity. Bars represent phylogenetic tree branches, the diamond represents the observed value, and the y-scale is frequency. [file 42523_2020_34_MOESM5_ESM.tif]

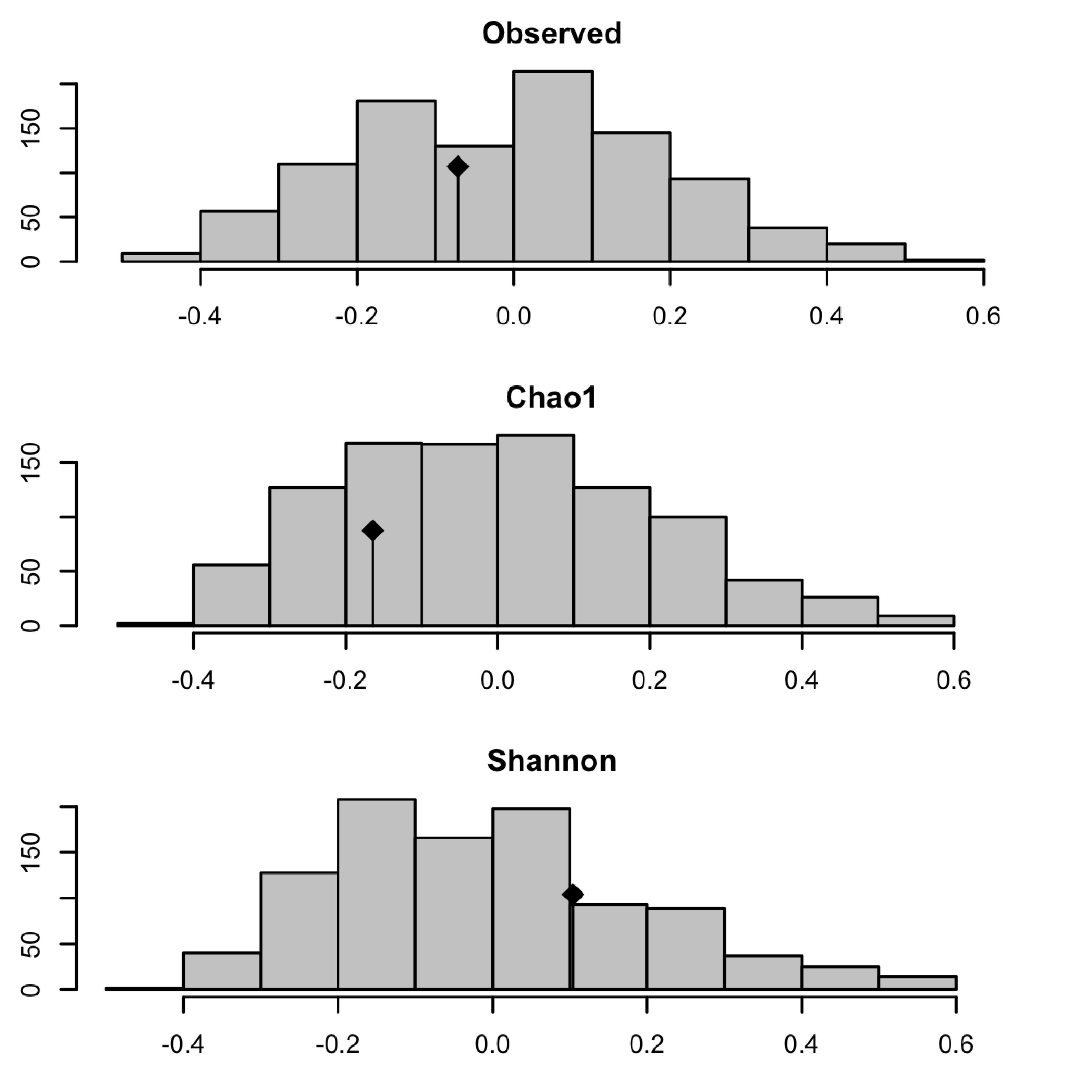

Supplement: Supplementary file 6 — Additional file 6: Fig. S2 Graphical representation of Abouheif’s Cmean calculations for alpha diversity. Bars represent phylogenetic tree branches, the diamond represents the observed value, and the y-scale is frequency. [file 42523_2020_34_MOESM6_ESM.png]

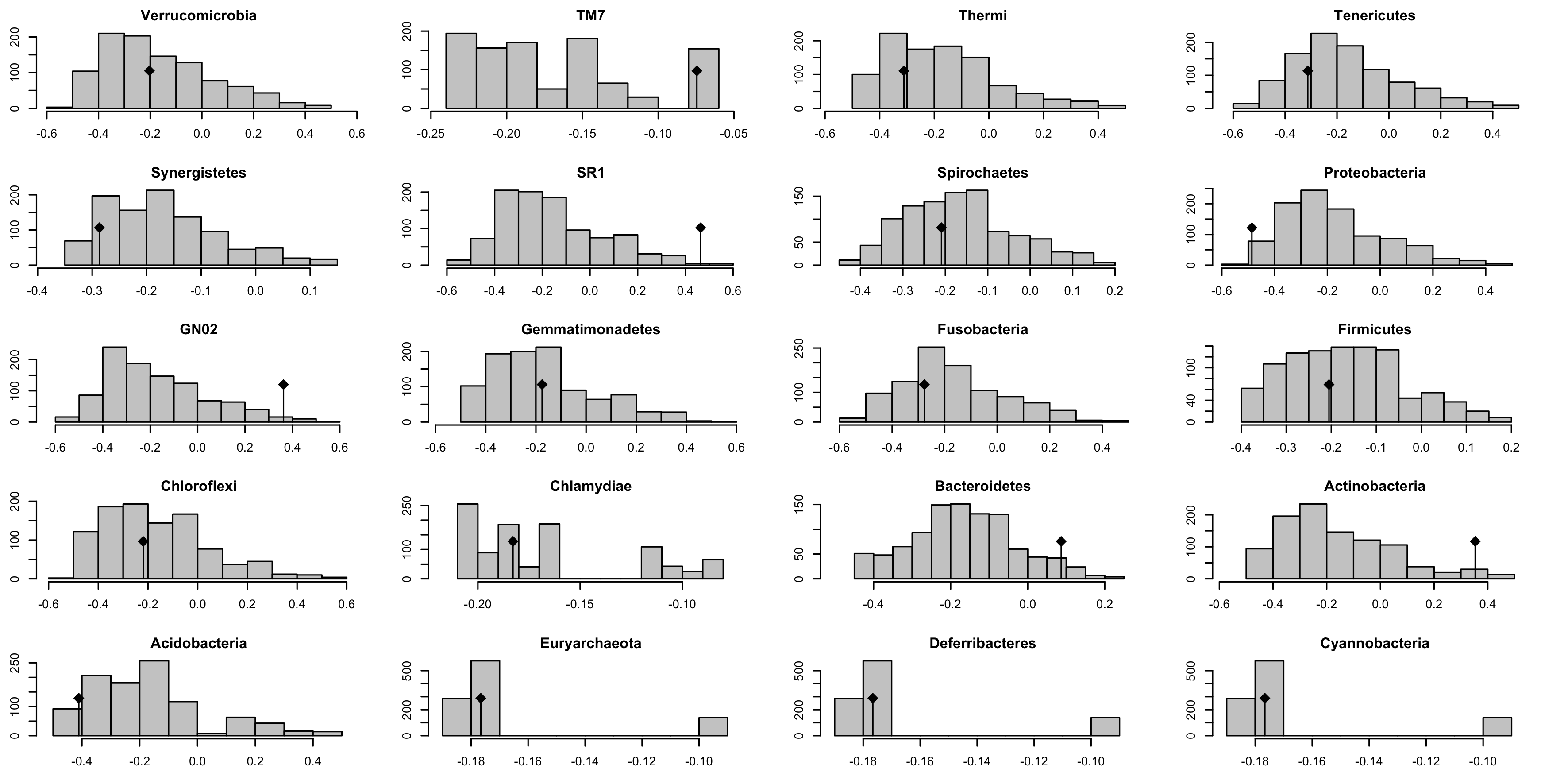

Supplement: Supplementary file 8 — Additional file 8: Fig. S3 Graphical representation of Moran’s I calculations for microbial composition. Bars represent phylogenetic tree branches, the diamond represents the observed value, and the y-scale is frequency. [file 42523_2020_34_MOESM8_ESM.tif]
