# Supplementary material for: Microbial symbiosis and coevolution of an entire clade of ancient vertebrates: the gut microbiota of sea turtles and its relationship to their phylogenetic history
Source: Anim Microbiome. 2020 May 7;2:17. doi: 10.1186/s42523-020-00034-8 (PMC7807503; doi:10.1186/s42523-020-00034-8)
Supplement: Supplementary file 7 — Additional file 7: Table S7. Moran’s I and Abouheif’s Cmean calculations for bacterial phyla and their correlation to sea turtle phylogeny. Significant values are indicated by bold text. [file 42523_2020_34_MOESM7_ESM.docx]

**Additional File 7**

**Table S7.**

| Phylum | Moran’s *I* | | | | Abouheif’s C_mean_ | | | |
| --- | --- | --- | --- | --- | --- | --- | --- | --- |
|  | Obs | Std.Obs | Alter | p | Obs | Std.Obs | Alter | p |
| Verrucomicrobia | -0.20 | -0.17 | greater | 0.48 | -0.03 | -0.16 | greater | 0.51 |
| TM7 | -0.07 | 1.72 | greater | 0.13 | 0.42 | 2.46 | greater | 0.06 |
| Thermi | -0.31 | -0.70 | greater | 0.71 | -0.16 | -0.80 | greater | 0.76 |
| Tenericutes | -0.31 | -0.74 | greater | 0.77 | -0.19 | -0.96 | greater | 0.79 |
| Synergistetes | -0.29 | -1.11 | greater | 0.87 | -0.22 | -1.19 | greater | 0.95 |
| SR1 | 0.46 | 2.92 | greater | **0.01** | 0.52 | 2.56 | greater | **0.01** |
| Spirochaetes | -0.21 | -0.33 | greater | 0.60 | -0.07 | -0.44 | greater | 0.62 |
| Proteobacteria | -0.49 | -1.64 | greater | 0.99 | -0.39 | -1.98 | greater | 1.00 |
| GN02 | 0.36 | 2.54 | greater | **0.01** | 0.42 | 2.21 | greater | **0.02** |
| Gemmatimonadetes | -0.18 | -0.05 | greater | 0.48 | 0.01 | -0.04 | greater | 0.47 |
| Fusobacteria | -0.28 | -0.57 | greater | 0.70 | 0.03 | 0.18 | greater | 0.42 |
| Firmicutes | -0.20 | -0.30 | greater | 0.59 | -0.12 | -0.64 | greater | 0.69 |
| Chloroflexi | -0.22 | -0.26 | greater | 0.53 | -0.02 | -0.20 | greater | 0.55 |
| Chlamydiae | -0.18 | -0.42 | greater | 0.52 | -0.02 | -0.13 | greater | 0.30 |
| Bacteroidetes | 0.09 | 1.84 | greater | 0.05 | 0.15 | 0.80 | greater | 0.22 |
| Actinobacteria | 0.35 | 2.54 | greater | **0.03** | 0.46 | 2.24 | greater | **0.02** |
| Acidobacteria | -0.41 | -1.23 | greater | 0.94 | -0.24 | -1.15 | greater | 0.87 |
| Euryarchaeota | -0.18 | -0.32 | greater | 0.42 | -0.13 | -0.75 | greater | 1.00 |
| Deferribacteres | -0.18 | -0.32 | greater | 0.42 | -0.13 | -0.75 | greater | 0.87 |
| Cyannobacteria | -0.18 | -0.32 | greater | 0.42 | -0.13 | -0.75 | greater | 1.00 |
